# Validation of dose‐volume calculation accuracy for intracranial stereotactic radiosurgery with volumetric‐modulated arc therapy using analytical and clinical treatment plans

**DOI:** 10.1002/acm2.70235

**Published:** 2025-08-21

**Authors:** Ioanna Grammatikou, Alexandra Drakopoulou, Antigoni Alexiou, Georgios Pissakas, Pantelis Karaiskos, Vasiliki Peppa

**Affiliations:** ^1^ Medical Physics Laboratory Medical School National and Kapodistrian University of Athens Athens Greece; ^2^ Department of Radiotherapy Alexandra Hospital Athens Greece

**Keywords:** dose grid, DVH accuracy, intracranial SRS, slice thickness, VMAT

## Abstract

**Background:**

Stereotactic radiosurgery (SRS) poses challenges in calculating dose volume histograms (DVHs) due to the inherent spatial discretization uncertainties.

**Purpose:**

To develop a procedure for quantifying discretization errors and assessing DVH accuracy in intracranial SRS applications.

**Methods:**

The capability of Monaco Treatment Planning System (TPS) to calculate structure and isodose volumes using slice thickness (ST) and dose grid (DG) of 1 mm was validated against analytical values, and in‐house calculations performed for 15 patients with brain metastases diameters ranging from 6 to 30 mm treated with a VMAT technique. For these patients, STs of 1.5 and 2 mm, and DGs of 2 and 3 mm were also explored to establish clinically acceptable thresholds, using isodose volume calculations and clinically relevant DVH indices.

**Results:**

Monaco TPS presented an excellent performance in calculating structure and isodose volumes for the 1‐mm ST and DG, with an average percentage difference compared to analytical and in‐house calculations within 2.1%. For the clinical treatment plans, switching to 2 and 3‐mm DGs led to statistically significant differences compared to 1‐mm DG across all the considered indices, yet the variations between the 1 and 2‐mm DGs remained under 5% when target diameters exceeded 20 mm. Although no statistical differences were observed between the calculated indices when different STs were considered, clinically significant differences were observed in selected cases with lesion diameters smaller than 20 mm.

**Conclusion:**

Monaco TPS demonstrated excellent performance in calculating structure and isodose volumes pertinent to SRS applications using 1‐mm ST and DG, while adherence to 1‐mm ST and DG should be maintained in clinical cases, unless target diameters surpass 20 mm, where STs and DGs up to 2 mm could be also utilized. The method developed in this study could act as a quality assurance procedure in order to establish clinically relevant discretization thresholds for SRS platforms.

## 1 INTRODUCTION

The dose volume histogram (DVH)^1^, ^2^ is a standard tool for assessing treatment plan quality in clinical practice^3^, ^4^ and for compiling data to support clinical trials.^5^ The accuracy in calculating clinically relevant metrics from DVH in radiotherapy treatment planning depends on how each treatment planning system (TPS) calculates structure and isodose volumes.^6^, ^7^, ^8^ Uncertainties in DVH calculation primarily arise from the 3D representation of the 2D contour‐based structures, the structure end‐capping, the interslice interpolation, as well as the spatial resolution of the dose grid.^7^

Previous studies have investigated the effect of slice thickness on target and critical organs volumes,^9^, ^10^, ^11^ the impact of calculation grid size^12^, ^13^, ^14^, ^15^, ^16^ and slice thickness^11^, ^17^ on DVH accuracy, as well as the DVH variability among different TPSs,^6^, ^8^, ^9^, ^18^, ^19^ showing that variations up to 30% may occur in specific clinical scenarios such as brachytherapy applications, where steep dose gradients exist.^14^ Although the impact of spatial discretization on DVH accuracy is expected to be severe in intracranial stereotactic radiosurgery (SRS) due to the high conformality to the target, steep dose gradients and the presence of very small structures, the literature on this topic is scarce focusing solely on the dosimetric impact of dose grid size,^9^, ^18^, ^20^, ^21^ while, to our knowledge, the influence of slice thickness on dosimetric accuracy has not been studied. While 1 mm is the most commonly used computed tomography (CT) slice thickness in intracranial SRS clinical practice, recommendations on this topic remain relatively ambiguous, with suggestions advocating for slice thicknesses up to 1 ,^22^ 1.25,^23^ or 1.5 mm.^24^ In order to determine limits suitable for each SRS platform however, the impact of slice thickness on DVH accuracy has to be quantified, while the potential use of thicker slice thicknesses that could reduce delineation time when large lesions are involved warrants investigation. Additionally, while consensus recommendations exist for dose grid size in SRS applications, suggesting the use of 1–2 mm depending on lesion size,^22^, ^23^, ^25^ these guidelines are somewhat vague and their uncritical adoption could lead to significant dosimetric errors. Moreover, given the long computational times required when using finely discretized calculation grids,^15^ alternative dose grid sizes could be explored, especially for larger metastases, to establish clinically related compromises between computational time and dosimetric accuracy.

Despite growing recognition of the need for a thorough review of calculation methodologies and standardization in the SRS era,^18^ specific guidance on testing procedures and acceptable performance levels for DVH calculations remains relatively understudied.^7^, ^9^ Stanley et al.^9^ developed a benchmark ground truth dataset, intended for the commissioning of a new SRS platform to facilitate the analysis of DVH calculations, that includes analytically derived Digital Imaging and Communications in Medicine (DICOM) structure and dose files. Although they acknowledge the incorporation of clinically relevant DVH indices beyond standard dosimetric and volumetric parameters in assessing DVH calculation accuracy, their evaluation in real clinical scenarios, where the shape, number, and proximity of the brain metastases could potentially yield more complex dose gradient distributions, has yet to be investigated in order to set appropriate thresholds.

The aim of this work was to develop a procedure for validating DVH accuracy in intracranial SRS applications using analytical ground truth and clinical data along with physical and clinically oriented indices, which could serve both as a commissioning procedure for new adopters of SRS platforms and as a pilot study that would enable end‐users to establish clinically relevant thresholds that reconcile dose calculation accuracy and time. This method was applied to Monaco (Elekta AB, Stockholm, Sweden) TPS, a platform commonly used for SRS applications,^26^, ^27^ for which the impact of discretization errors on DVH accuracy has not yet been explored within the context of SRS.

## 2 METHODS

### 2.1 TPS validation

TPS validation in calculating structure and isodose volumes relevant to SRS applications was conducted at a single institution using the Monaco 6.1.2.0 TPS, which is the software available for treatment planning and DVH analysis. This process consisted of two steps, with the first involving analytical geometries and dose distributions that mimic real SRS cases, and the second using actual clinical cases. For the analytical‐based geometries and dose distributions, TPS calculations were compared to their corresponding known nominal values. For the clinical cases, an in‐house program was developed to generate the reference dataset for validating TPS results, which was initially benchmarked against the nominal values of the analytical dataset used in the first step. This method follows the recommendation by Nelms et al.,^7^ suggesting that DVH calculations with the highest accuracy against analytical geometries can serve as a reference dataset for benchmarking clinically based volume and isodose calculations, where nominal values are unavailable.

#### 2.1.1 Analytical structures and dose distributions

The accuracy of Monaco TPS in calculating structure and isodose volumes pertinent to intracranial SRS applications was initially evaluated using the analytically derived ground truth dataset prepared by Stanley et al.^9^ Validation was performed for dose grid (DG) and slice thickness (ST) values of 1 mm recommended for LINAC SRS applications of multiple brain metastases^22^, ^28^ and utilized in our department for relevant clinical cases. The synthetic CT datasets with a voxel size of 0.6 × 0.6 × 1 mm^3^, along with the set of RT structure files including 50 spheres of equal diameters each, ranging from 3 to 20 mm, the corresponding RT plan files and the analytical RT dose matrices with a resolution of 1 × 1 × 1 mm^3^ that were developed for linear accelerator‐based SRS, were imported to Monaco TPS. Following the procedure presented by Stanley et al.,^9^ the volume of each sphere reported by the TPS, as well as the volumes encompassed by the isodose levels of 100% (V_100%_) and 50% (V_50%_) were obtained through the DVH analysis tool, using the evaluation volumes included in the RTSS file around each sphere, where the dose is greater than or equal to 25% of the dose at the target sphere. The TPS‐based results were compared to the corresponding nominal values calculated using the expression $\frac{\pi D^{3}}{6}$ for the structure volumes, and the analytical equation for the dose distribution defined by Stanley et al.^9^ for given distances from the center of the spheres.

#### 2.1.2 Clinical cases

The capability of Monaco TPS to accurately calculate structure and isodose volumes in SRS applications was further evaluated in real clinical scenarios to determine if the results from the synthetic geometries are applicable to actual cases. In specific, 15 patients treated for multiple brain metastases at the moment this study received approval from our institutional review board were retrospectively selected in this study. The current patient sample was selected, as it is consistent with similar studies in the literature^11^, ^14^, ^16^, ^20^, ^21^ and aligns with sample sizes commonly used in pilot studies addressing small to large standardized effect sizes (*d* ≥ 0.1).^29^ For each patient, the CT scan was acquired with a 1‐mm ST, while a volumetric‐modulated arc therapy (VMAT) treatment plan designed for the Infinity Linac (Elekta AB, Stockholm, Sweden) equipped with the Agility MLC (Elekta AB, Stockholm, Sweden) of 160 leaves (spatial resolution of 5 mm at isocenter) was generated utilizing a single isocenter along with five non‐coplanar arcs. Dose prescription involved 30 Gy in 5 fractions, aiming at least 95% coverage of the planning target volumes (PTVs) by the prescribed dose. Dose to medium in medium (D_m,m_) was calculated using the XVMC Monte Carlo dose engine^30^, ^31^ employing an isotropic DG of 1 mm. In total, 68 PTVs were investigated, which were categorized into six groups according to the maximum PTV diameter measured on the axial CT slices (Table 1). It should be noticed that target diameters larger than 6 mm were included in this study, as targets smaller than 5 mm are excluded from treatment in our clinic. This exclusion is due to the challenges in generating highly conformal dose distributions with the 5‐mm MLC, which may lead to increased volumes of normal brain receiving dose above the prescribed dose and a higher risk of brain necrosis. The PTV volumes reported by the TPS, along with the volumes encompassed by the isodose levels of 110%, 100%, 98%, 95%, 80%, and 50%, hereinafter referred to as V_dose_, were considered as the validation parameters. The V_dose_ values were calculated for each PTV using the DVH analysis tool of Monaco TPS along with evaluation shells of 1.5 cm width generated around each target, ensuring that no dose contribution from PTVs outside the evaluation shell was included. It should be noticed that dose was normalized such that the 100% isodose level coincided with the prescribed dose.

**TABLE 1:** PTV volumes of the patient cohort calculated from the CT images of 1‐mm ST.

	Volume (cm^3^)
PTV diameter (mm)	Median [mix, max]
6	0.52 [0.24, 0.65]
10	1.11 [0.73, 1.41]
15	2.47 [1.78, 2.93]
20	5.17 [2.86, 6.44]
25	7.03 [4.36, 9.16]
30	12.31 [6.49, 13.95]

In order to generate reference numerical values for the PTV volumes and V_dose_ parameters, where analytical data cannot be produced, an in‐house program was developed in MATLAB (MATLAB R2020b, The MathWorks Inc., Natick, Massachusetts, USA) using the function *inpolygon*,^32^ which was initially benchmarked against the analytical structure and isodose volumes prepared by Stanley et al.^9^ Validation of the TPS for the clinical cases involved investigating the correlation between the calculated TPS‐based PTV and V_dose_ volumes with the corresponding results for the 1‐mm ST and DG obtained from the in‐house program through the application of a linear fit.

### 2.2 Effect of DG size and ST on SRS clinical indices

The treatment plans of the fifteen patients with multiple brain metastases, acquired using 1 mm ST and 1 mm DG were used to validate the TPS and also served as the reference datasets for investigating the impact of DG and ST on commonly used SRS plan quality indices. Specifically, the original treatment plans were recalculated for each patient using isotropic DGs of 2 and 3 mm. Although the 3‐mm DG departs significantly from the 1‐mm DG recommended for single isocenter LINAC SRS applications of multiple brain metastases,^28^ it was mainly considered in this work for its suitability investigation when larger metastases are involved. In order to evaluate the impact of ST on plan quality indices, the clinical CT images of each patient acquired with the 1‐mm ST were resampled to 1.5 and 2‐mm STs, maintaining their in‐plane resolution. It should be noticed that a common origin, defined by the Image Position Patient field in the DICOM header, was used for the three CT image datasets of each case, in an effort to minimize PTV misalignments among the different datasets. Similar to the DGs investigated in this work, the 2‐mm ST was selected to assess whether it could act as a dosimetrically equivalent surrogate for larger metastases. For each patient, the original PTVs were copied to the CT image sets of 2 and 3‐mm STs and the treatment plan was recalculated using a 1‐mm DG.

The effect of DG and ST was initially investigated on the V_dose_ parameters employed for the clinically‐based TPS validation, by calculating the volumes encompassed by the isodose levels of 110%, 100%, 98%, 95%, 80%, and 50% for each PTV. To this end, the ratios of the V_dose_ parameters acquired with the alternative STs or DGs ($V_{dose(eval)})\;$ to the corresponding reference values obtained with the 1‐mm ST and DG ($V_{dose(ref)})$ were evaluated and correlated with the $V_{dose(ref)}$ using the Spearman's rank correlation coefficient and the corresponding *p*‐value. The impact of DG and ST on SRS plan quality was further assessed through the minimum percentage of the prescribed dose delivered to 95% of the PTV, D_95_, the percentage of the PTV receiving dose greater than the prescribed dose, V_30Gy_, the Conformity Index (CI),^33^ and the Gradient Index (GI),^34^ as described in the following formulas:

(1)

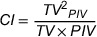

(2) $$GI=\frac{V_{50\%\;Rx}}{V_{100\%\;Rx}}$$

where, $TV_{PIV}$ is the volume of the target covered by the prescription dose, TV is the target volume, PIV is the prescription isodose volume, $V_{50\%\;Rx}$ is the volume of the 50% isodose line, and $V_{100\%\;Rx}$ is the volume of the 100% isodose line. For both CI and GI, the aforementioned isodose volumes were manually calculated for each PTV through the DVH analysis tool using the evaluation shells of 1.5 cm width around each target that encompass both $V_{50\%\;Rx}$ and $V_{100\%\;Rx}$ isodose volumes. It should be noticed that the formula described in Equation 1 was used in this study to calculate CI, rather than the one used by the TPS, to facilitate comparison with the literature.

The calculated indices associated with the altered DGs and STs were statistically compared to those obtained with the 1‐mm DG and ST, using the Wilcoxon signed‐rank test with a significance criterion of *p* ≤ 0.05. To strengthen the validity of the results given the relatively small sample size considered, a statistical power analysis was performed using G*Power 3.1.9.7 (Heinrich Heine Universität, Düsseldorf, Germany). The effect size *d* and the required total sample size were calculated for a significance level (*α*) of 0.05 along with a statistical power (*1 – β*) of 0.8.

## 3 RESULTS

### 3.1 TPS validation

Table 2 summarizes the average percentage differences and range of the TPS and in‐house calculations with the corresponding analytical values derived for the structure volumes, the V_100%_ and V_50%_ indices (($\frac{Indice_{TPS/in-house}}{Indice_{analytical}}-1)\times 100)$. For target diameters larger than 7 mm, the absolute average percentage differences between the Monaco TPS and the corresponding analytical values for the structure volumes decreased as the sphere diameter increased, ranging from 0.3% for the 7 mm target to 0.0% for the 20 mm target. A similar pattern was observed for the isodose volumes, with the absolute average percentage differences for the V_100%_ and V_50%_ decreasing from 0.2% to 0.1% and from 0.1% to 0.0%, respectively, as the diameter increased from 7 to 20 mm. For the 3‐mm diameter targets, the average and maximum percentage differences in the calculated structure volumes were equal to 2.1% and 8.0%, respectively. Although differences down to ‐23.7% were recorded for the V_100%_ of the 3‐mm diameter spheres, the average percentage differences in the calculated isodose volumes remained within 0.8%. A corresponding pattern was evident between the in‐house calculations and analytical results with variations increasing as target size decreased. For target diameters larger than 7 mm, the absolute average percentage differences between the in‐house program and the corresponding analytical values ranged from 0.2% to 0.0% for the structure volumes, from 0.0% to 0.1% for the V_100%_ and from 0.0% to 0.0% for the V_50%_, as the target diameter increased from 7 to 20 mm. For the structure volumes of the 3‐mm diameter spheres, the average and maximum absolute percentage differences were equal to 1.4% and 6.5%, respectively, while the corresponding differences for the V_100%_ were 0.7% and 10.9%, respectively. The close agreement observed between the in‐house and analytical calculations for the ground truth dataset, which was better than the agreement discerned between the TPS and analytical‐based results, renders the in‐house program suitable for validating clinically based structure and isodose volumes in SRS applications, where reference data do not exist.^7^

**TABLE 2:** Comparisons performed for the ground truth datasets in terms of the average difference and range between the structure volume, V_100%_ and V_50%_ calculated using the TPS or the in‐house routine and the corresponding analytically derived values for each target size.

	TPS validation				In‐house program validation	
	mean ΔV (Range) [%]					
Target diameter (mm)	Target Volume	V_100%_	V_50%_	Target Volume	V_100%_	V_50%_
3	2.14 (‐8.04, 6.10)	0.83 (‐23.72, 17.89)	0.17 (‐1.14, 2.84)	1.35 (‐5.33, 6.50)	0.69 (‐9.78, 10.89)	0.00 (‐1.30, 2.52)
7	−0.31 (‐2.00, 1.90)	0.15 (‐4.85, 4.11)	0.11 (‐0.53, 0.94)	−0.18 (‐2.98, 2.43)	0.00 (‐3.41, 3.11)	0.00 (‐0.65, 0.88)
10	0.07 (‐1.07, 0.65)	0.05 (‐2.38, 1.43)	0.11 (‐0.46, 0.90)	0.03 (‐1.27, 0.86)	0.00 (‐2.25, 1.13)	0.00 (‐0.43, 0.73)
15	−0.02 (‐0.40, 0.22)	0.06 (‐0.67, 1.20)	0.08 (‐0.46, 0.36)	−0.01 (‐0.35, 0.28)	0.00 (‐0.78, 1.08)	0.00 (‐0.53, 0.28)
20	0.02 (‐0.23, 0.15)	0.10 (‐0.53, 0.35)	0.04 (‐0.11, 0.38)	0.02 (‐0.15, 0.16)	0.05 (‐0.55, 0.33)	−0.03 (‐0.10, 0.35)

**FIGURE 1:**
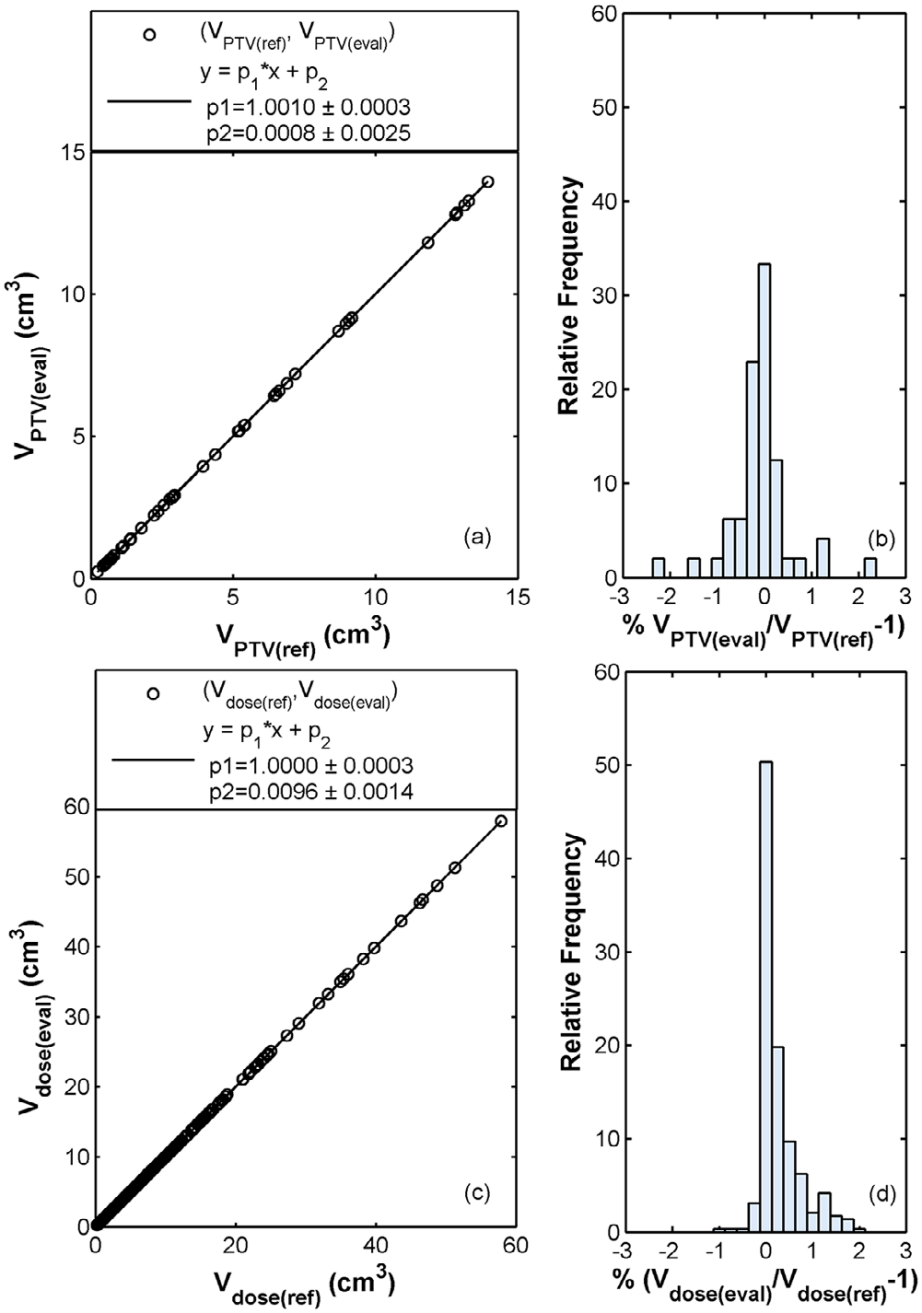
Plots of the (a) *V_PTV(eval)_* versus *V_PTV(ref)_* and (c) *V_dose(eval)_* versus *V_dose(ref)_* obtained from the TPS (eval) and the in‐house program (ref) for the 1‐mm ST and DG along with the results of the linear fit performed on both datasets across all patients (*R^2^* = 1). Relative local dose difference histograms between the (b) *V_PTV(eval)_* and *V_PTV(ref)_* and (d) *V_dose(eval)_* and *V_dose(ref)_* are also presented.

Figure 1a illustrates the comparison between the PTV volumes calculated using the TPS and the in‐house program across all patients for the 1‐mm ST, using the latter as the reference dataset. Results of the linear fit equation $y=p_{1}\;\times x+p_{2}$ demonstrated an excellence performance for the TPS‐based structure volumes with an *R*‐squared value of 1, $p_{1}=1.0010\pm 0.0003\;$ and $p_{2}=0.0008\pm 0.0025$. Similarly, corresponding comparisons in Figure 1c between the TPS and in‐house V_dose_ parameters for the 1‐mm DG and ST revealed a perfect linear correlation, with an *R*‐squared value of 1, $p_{1}=1.0000\pm 0.0003\;$ and $p_{2}=0.0096\pm 0.0014$. These findings align with the percentage differences calculated between the TPS and in‐house results (Figure 1b, d), with 100% of the investigated PTV volumes and 100% of the considered isodose volumes demonstrating an agreement within ± 3%. It should be noticed that the observed increased TPS accuracy was also evident when calculating the 1‐mm ST and DG‐based PTV and isodose volumes smaller than 1 cm^3^, where the percentage differences with the corresponding reference results remained within 2.3% and 1.7%, respectively.

### 3.2 Effect of ST and DG size on isodose volumes

**FIGURE 2:**
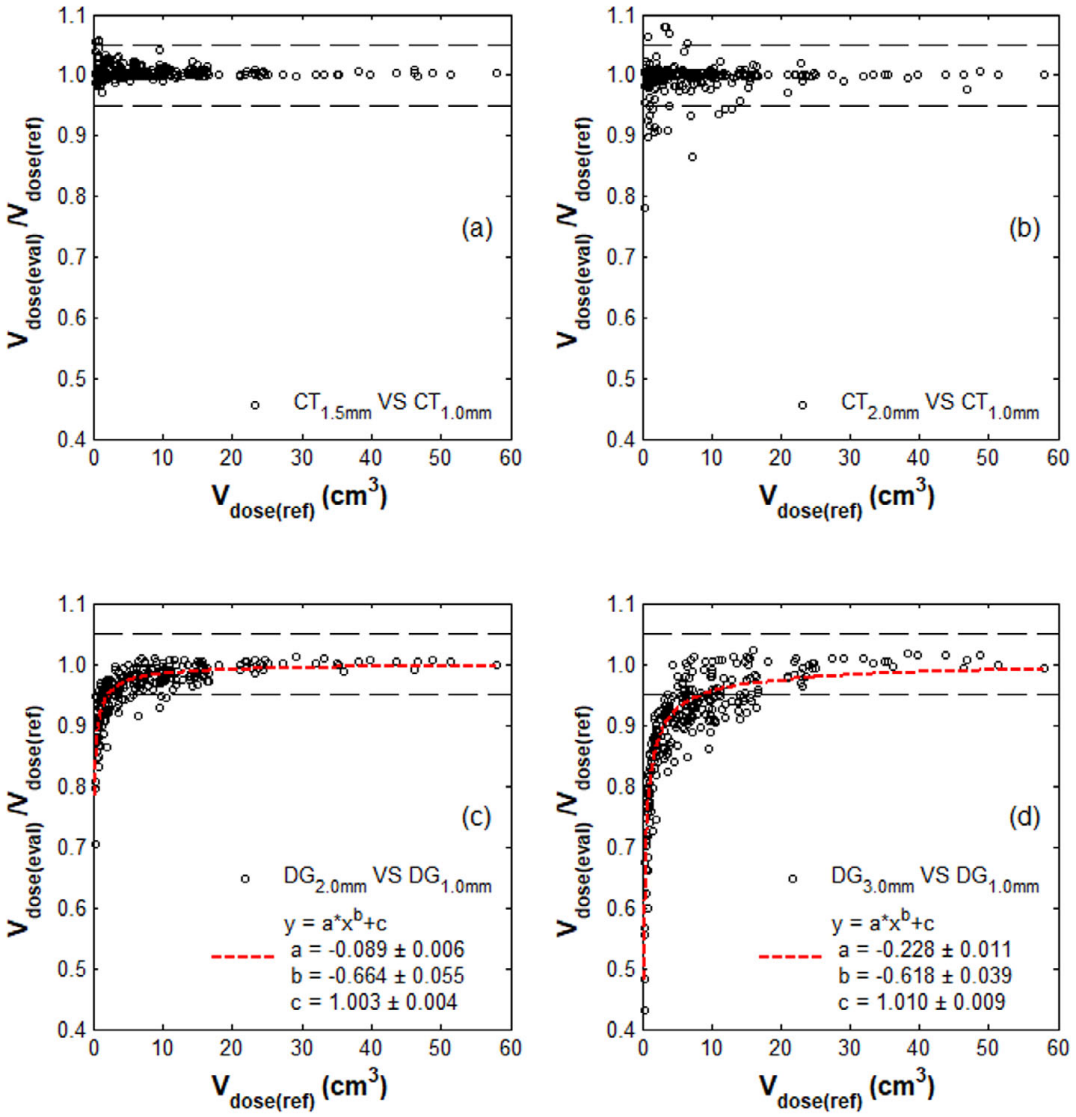
Ratios of $V_{\mathrm{dose}(\mathrm{eval})}/V_{\mathrm{dose}(\mathrm{ref})}$ calculated across the patient cohort for the (a) 1.5‐mm and (b) 2‐mm STs (eval) with respect to 1‐mm ST (ref), and (c) 2‐mm and (d) 3‐mm DGs (eval) with respect to 1‐mm DG (ref), as a function of $V_{\mathrm{dose}(\mathrm{ref})}$. Results of the power fit performed on datasets DG_2.0 mm_ VS DG_1.0 mm_ and DG_3.0 mm_ VS DG_1.0 mm_ are also presented. The corresponding normalized root mean square errors (NRMSE), calculated relative to the range of the $V_{\mathrm{dose}(\mathrm{eval})}/V_{\mathrm{dose}(\mathrm{ref})}$ values, are equal to 0.067 and 0.061, respectively.

Figure 2a, b present the ratios of $V_{dose(eval)}/V_{dose(ref)}\;$ calculated for the 1.5 and 2‐mm STs with respect to $V_{dose(ref)}$ calculated for the 1‐mm ST. The calculated isodose volumes yielded a weak correlation between the $V_{dose(eval)}/V_{dose(ref)}$ and the $V_{dose(ref)}$ for both STs (*ρ* < 0.04, *p* > 0.10), with resultant median values for the $V_{dose(eval)}/V_{dose(ref)}$ ratios of 1.003 (range [0.972,1.059]) and 1.000 (range [0.782,1.080]) for the 1.5 and 2‐mm STs, respectively. Differences exceeding 5% between the 1.5‐mm ST‐based $V_{dose(eval)}$ and $V_{dose(ref)}$ were identified for isodose volumes smaller than 1 cm^3^, corresponding to isodose diameters less than 12 mm. These differences account for 9.4% of the calculated data with $V_{dose(ref)}\;$ smaller than 1 cm^3^. A similar pattern was observed when comparing the isodose volumes between the 2 and 1‐mm STs, with 10.6% of the data for $V_{dose(ref)}$ smaller than 7 cm^3^, corresponding to isodose diameters less than 24 mm, showing differences greater than 5%.

In Figure 2c, d, the ratios of $V_{dose(eval)}/V_{dose(ref)}\;$ calculated for the 2 and 3‐mm DGs are significantly lower than 1 for small $V_{dose(ref)}$ values, yet they present an increase toward 1 as the $V_{dose(ref)}$ increases, resulting in *ρ* coefficients of 0.53 (*p* < 0.001) and 0.58 (*p* < 0.001), respectively. The applied fitting equation $y=a\times x^{b}+c$ revealed that the $V_{dose(eval)}/V_{dose(ref)}$ values calculated with the 2 and 3‐mm DGs were higher than 0.95 for isodose volumes larger than 2 cm^3^ (isodose diameters greater than 16 mm) and 10 cm^3^ (isodose diameters greater than 27 mm), respectively, while they decreased rapidly for smaller isodose volumes.

### 3.3 Effect of ST and DG size on SRS clinical indices

The results for V_30Gy_, CI, and GI calculated from DVHs for the 1, 1.5, and 2 mm STs are presented in Table 3. For the vast majority of DVH indices calculated using the 1.5 and 2‐mm STs, the results were statistically equivalent to those acquired with the 1‐mm ST, in accordance with the weak correlation observed between the $V_{dose(eval)}$ and $V_{dose(ref)}$ parameters. Despite the statistical equivalence between the different STs however, substantial percentage differences greater than 5% were observed in the calculated median CI values for the 10‐mm PTV diameter. Similar differences were found in the median GI values for the 15‐mm PTV diameter when comparing the 2 and 3‐mm STs to the 1‐mm ST. These findings are in accordance with the results presented in Figure 2a and b. The V30Gy showed generally smaller discrepancies in the calculated median values across the varying STs compared to the CI and GI. The maximum percentage difference of ‐2.8% was observed between the 2 and 1‐mm STs for the 6‐mm PTV diameter. For the PTV diameters exceeding 20 mm, percentage differences in the calculated median DVH indices of the 1.5 and 2‐mm STs with the 1‐mm ST were lower than 5%. The results for D_95_ calculated for the 1, 1.5, and 2‐mm STs are presented in Table 4. Similar to the other DVH indices investigated, the D_95_ parameters obtained with the 1.5 and 2‐mm STs were statistically equivalent to those derived from the 1‐mm ST for all PTV sizes. The differences in the median D_95_ values decreased with increasing PTV size, while they were consistent with the corresponding differences in V_30Gy_, with a maximum difference of ‐2.2% observed between the 2 and 1‐mm STs of the 6‐mm PTV diameter. Results of the statistical power analysis generally yielded required sample sizes smaller than 15, except for specific instances shown in Tables 3 and 4, which correspond to *p*‐values greater than 0.95. In these cases, an effect size of approximately 0.2 was obtained. This small effect size, however, was attributed to the presence of an extreme outlier in the percentage difference data. Upon exclusion of this outlier, the calculated effect size increased to over 0.58, while the required sample size decreased to fewer than 14 participants.

**TABLE 3:** Comparison of the DVH parameters derived using 1, 1.5, and 2‐mm ST for each PTV size of the patient cohort.

		V_30Gy_ (%)			CI			GI		
PTV diameter (mm)	CT (mm)	Median	[min, max]	*p*	Median	[min, max]	*p*	Median	[min, max]	*p*
6	1.0	98.49	[95.10, 100.0]		0.63	[0.43, 0.81]		7.81	[6.28, 9.89]	
	1.5	97.19	[91.29, 99.65]	0.84	0.62	[0.45, 0.77]	0.77	7.83	[6.18, 9.83]	0.84
	2.0	95.71	[92.74, 99.47]	1.00	0.64	[0.44, 0.75]	0.58	8.60	[6.28, 9.65]	0.11
10	1.0	97.55	[95.08, 98.73]		0.71	[0.48, 0.85]		6.18	[5.36, 7.65]	
	1.5	97.52	[94.31, 99.28]	0.46	0.75	[0.51, 0.85]	1.00	6.21	[5.33, 7.74]	0.15
	2.0	96.76	[94.87, 98.61]	0.08	0.74	[0.49, 0.84]	0.30	6.37	[5.36, 7.57]	0.11
15	1.0	96.75	[96.38, 98.62]		0.78	[0.73, 0.82]		4.74	[4.17, 5.73]	
	1.5	97.09	[92.24, 98.07]	0.74	0.79	[0.75, 0.85]	0.84	4.98	[4.36, 5.70]	0.84
	2.0	97.18	[91.67, 98.39]	0.74	0.77	[0.70, 0.82]	0.01	4.48	[4.20, 5.80]	0.67
20	1.0	95.93	[95.04, 97.84]		0.84	[0.76, 0.87]		4.25	[3.76, 5.16]	
	1.5	96.71	[95.25, 97.75]	0.38	0.85	[0.75, 0.88]	0.84	4.18	[3.72, 5.13]	0.98
	2.0	96.73	[95.94, 97.39]	0.15	0.84	[0.76, 0.86]	0.38	4.26	[3.74, 5.16]	0.22
25	1.0	96.95	[95.03, 98.05]		0.85	[0.78, 0.88]		3.84	[3.28, 4.10]	
	1.5	96.63	[95.54, 97.47]	0.84	0.86	[0.80, 0.96]	0.31	3.84	[3.27, 4.08]	0.38
	2.0	96.51	[95.10, 97.11]	0.71	0.83	[0.77, 0.88]	0.02	3.73	[3.28, 4.10]	0.35
30	1.0	96.72	[94.99, 98.03]		0.87	[0.78, 0.95]		3.66	[3.17, 5.47]	
	1.5	96.52	[92.74, 97.72]	1.00	0.86	[0.80, 0.90]	0.64	3.66	[3.15, 5.49]	0.61
	2.0	95.50	[90.44, 97.76]	0.55	0.85	[0.78, 0.89]	0.02	3.65	[3.17, 5.35]	0.81

Statistical significance (*p* ≤ 0.05) was tested using a paired sample Wilcoxon signed‐rank test.

**TABLE 4:** Comparison of the D_95_ derived using 1, 2, and 3‐mm DG and 1, 1.5, and 2‐mm ST for each PTV size of the patient cohort.

	D_95_ (%)							
PTV diameter (mm)	DG (mm)	Median	[min, max]	*p*	CT (mm)	Median	[min, max]	*p*
6	1.0	103.39	[100.26, 105.54]		1.0	103.39	[100.26, 105.54]	
	2.0	101.96	[99.09, 104.48]	0.02	1.5	101.92	[97.16, 106.14]	0.19
	3.0	98.98	[95.53, 101.57]	0.01	2.0	101.21	[97.69, 107.69]	0.06
10	1.0	102.84	[100.89, 104.40]		1.0	102.84	[100.89, 104.40]	
	2.0	101.71	[99.56, 103.52]	0.01	1.5	101.97	[99.54, 105.80]	0.38
	3.0	99.59	[97.52, 101.24]	0.01	2.0	102.18	[99.91, 106.13]	0.74
15	1.0	101.38	[100.33, 105.96]		1.0	101.38	[100.33, 105.96]	
	2.0	100.62	[99.04, 104.06]	0.01	1.5	101.30	[98.47, 104.13]	0.15
	3.0	98.96	[98.31, 102.93]	0.01	2.0	100.65	[95.79, 103.86]	0.25
20	1.0	101.21	[100.06, 102.13]		1.0	101.21	[100.06, 102.13]	
	2.0	100.37	[99.05, 100.95]	0.01	1.5	101.12	[99.68, 102.50]	0.95
	3.0	98.97	[97.63, 99.53]	0.01	2.0	101.24	[99.96, 102.51]	0.46
25	1.0	101.38	[100.17, 103.65]		1.0	101.38	[100.17, 103.65]	
	2.0	100.46	[99.79, 102.92]	0.01	1.5	101.29	[99.93, 102.93]	0.65
	3.0	98.96	[98.20, 100.62]	0.01	2.0	101.18	[99.60, 102.57]	0.31
30	1.0	100.71	[99.99, 102.20]		1.0	100.71	[99.99, 102.20]	
	2.0	100.24	[99.91, 101.76]	0.02	1.5	100.30	[98.45, 102.66]	0.15
	3.0	99.36	[98.59, 100.26]	0.01	2.0	99.74	[98.16, 102.74]	0.11

Statistical significance (p ≤ 0.05) was tested using a paired sample Wilcoxon signed‐rank test.

**FIGURE 3:**
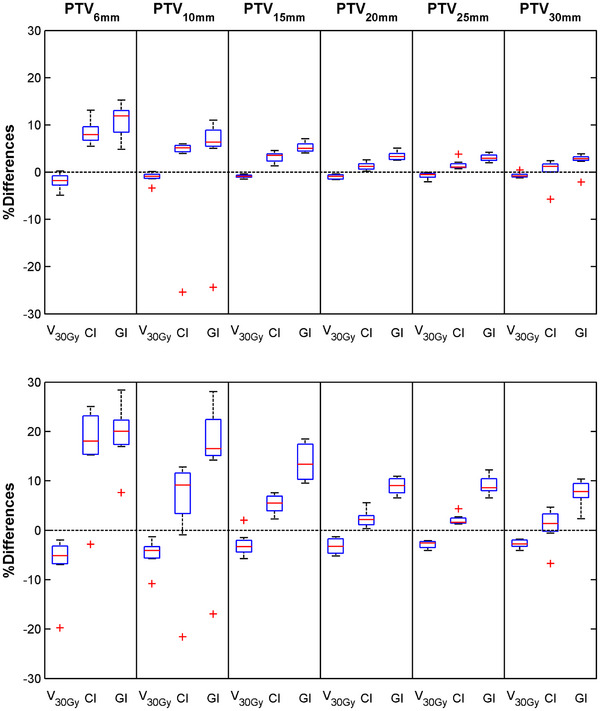
Box and whisker plots showing the local percentage differences of the considered indices 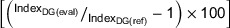 between the evaluated 2‐mm (upper) and 3‐mm (lower) DGs with the reference 1‐mm DG for each PTV size of the patient cohort.

The comparison of DVH results calculated with the 2 and 3‐mm DGs relative to 1‐mm DG is elaborated in Table 5. A general statistically significant underestimation can be seen for the V_30Gy_ calculated with the 2 and 3‐mm DGs compared to 1‐mm DG, in line with the corresponding differences observed for the isodose volumes. Conversely, the 2 and 3‐mm DGs presented a statistically significant overestimation of the CI and GI with respect to 1‐mm DG for all PTV sizes, except for the largest PTV where the CI was equivalent among the three datasets. These results agree with the percentage differences presented in Figure 3 between the 2 and 3‐mm DG‐based DVH indices and those calculated with the 1‐mm DG, which increase with decreasing PTV and increasing DG size. Overall, GI exhibited larger percentage differences compared to V_30Gy_ and CI, with median values between the 3 and 1‐mm DGs exceeding 5% for each PTV size. For the 2‐mm DG, median values of the percentage differences were lower than 5% for all DVH indices when the PTV diameter surpassed 20 mm, with maximum values lower than ‐0.9%, 1.2%, and 3.3% for the V_30Gy_, CI, and GI, respectively. Similar to the V_30Gy_, results for the D_95_ parameter associated with the 1 and 2‐mm DGs in Table 4 showed a statistically significant underestimation compared to 1‐mm DG, which was nevertheless consistently smaller than that observed for the V_30Gy_. This underestimation became more pronounced as the PTV size decreased, with a maximum difference in the median values of ‐4.4% discerned between the 2 and 1‐mm DGs of the 6‐mm PTV diameter. The statistical power analysis conducted on the data acquired with varying DG size indicated effect sizes greater than 1.03 and required sample sizes of fewer than 8 cases.

**TABLE 5:** Comparison of the DVH parameters derived using 1, 2, and 3‐mm DG for each PTV size of the patient cohort.

		V_30Gy_ (%)			CI			GI		
PTV diameter (mm)	DG (mm)	Median	[min, max]	*p*	Median	[min, max]	*p*	Median	[min, max]	*p*
6	1	98.49	[95.10, 100.0]		0.63	[0.43, 0.81]		7.81	[6.28, 9.89]	
	2	95.02	[93.19, 99.34]	0.02	0.68	[0.49, 0.86]	0.01	8.52	[6.98, 11.22]	0.01
	3	92.55	[79.30, 96.99]	0.01	0.74	[0.54, 0.79]	0.02	10.26	[7.53, 13.51]	0.01
10	1	97.55	[95.08, 98.73]		0.71	[0.48, 0.85]		6.18	[5.36, 7.65]	
	2	96.59	[93.80, 98.42]	0.02	0.75	[0.50, 0.89]	0.01	6.62	[5.62, 8.38]	0.01
	3	93.94	[86.32, 95.51]	0.01	0.79	[0.53, 0.86]	0.02	7.24	[6.26, 9.80]	0.01
15	1	96.75	[96.38, 99.53]		0.78	[0.73, 0.82]		4.74	[4.17, 5.74]	
	2	95.86	[95.41, 99.18]	0.01	0.81	[0.76, 0.86]	0.01	5.03	[4.36, 6.09]	0.01
	3	93.53	[92.15, 98.39]	0.02	0.83	[0.78, 0.86]	0.01	5.39	[4.57, 6.73]	0.01
20	1	95.93	[95.04, 97.84]		0.84	[0.76, 0.87]		4.25	[3.76, 5.16]	
	2	95.35	[93.59, 96.34]	0.01	0.85	[0.78, 0.89]	0.01	4.39	[3.85, 5.44]	0.01
	3	93.81	[89.97, 94.44]	0.01	0.86	[0.80, 0.89]	0.01	4.68	[4.01, 5.72]	0.01
25	1	96.95	[95.03, 98.05]		0.85	[0.78, 0.88]		3.84	[3.28, 4.09]	
	2	95.52	[94.24, 97.98]	0.01	0.86	[0.79, 0.89]	0.01	3.97	[3.36, 4.22]	0.01
	3	93.31	[92.41, 95.96]	0.01	0.87	[0.81, 0.89]	0.01	4.23	[3.53, 4.57]	0.01
30	1	96.72	[94.99, 98.03]		0.87	[0.78, 0.95]		3.66	[3.17, 5.47]	
	2	95.75	[94.84, 96.83]	0.04	0.88	[0.80, 0.90]	0.20	3.76	[3.25, 5.62]	0.04
	3	93.55	[92.55, 95.40]	0.01	0.88	[0.81, 0.89]	0.31	3.92	[3.42, 6.03]	0.01

Statistical significance (*p ≤ 0.05*) was tested using a paired sample Wilcoxon signed‐rank test.

## 4 DISCUSSION

A method was developed in this study to validate the capability of Monaco TPS to accurately calculate structure and isodose volumes in intracranial SRS applications involving multiple brain metastases, using ground truth and clinical data of patients treated with a VMAT technique. With the recommended ST and DG of 1 mm,^22^, ^28^ Monaco TPS exhibited a remarkable performance with average differences relative to reference results up to 2.1% and 0.3% for the analytical and clinical cases, respectively. These findings suggest that discretization of the computed dose distribution by Monaco TPS with the 1‐mm ST and DG can effectively capture the steep dose gradients, peaks, and valleys pertinent to analytical and realistic SRS applications.

The results for isodose volumes and DVH indices in the clinical cases obtained with varying ST demonstrated statistical equivalence. This equivalence is attributed to the lack of a clear pattern in the $V_{dose(eval)}/V_{dose(ref)}$ distribution relative to $V_{dose(ref)}$, where ratio values both higher and lower than 1 are nearly equally distributed around 1, rather than the absence of significant discrepancies. Clinically relevant differences higher than 5%^35^ were observed for the 1.5 and 2‐mm STs compared to 1‐mm ST in specific cases with isodose diameters smaller than 12 and 24 mm, respectively. Due to the unpredictable nature of the differences in isodose volumes and the resultant DVH indices with varying ST, which may be clinically relevant when PTV diameters are smaller than 20 mm, adherence to the 1‐mm ST should be maintained for small lesions. For PTV diameters larger than 20 mm, the use of 1.5 or 2‐mm STs could be a reasonable alternative to reach a compromise between DVH accuracy and volume delineation time.

DG appeared to have a more significant impact on isodose volume calculation accuracy compared to ST, particularly for dose diameters smaller than 16 and 27 mm for the 2 and 3‐mm DGs, respectively, while the $V_{dose(eval)}/V_{dose(ref)}$ distribution relative to $V_{dose(ref)}\;$ followed a power function. It should be noted, however, that the provided empirical equations should be applied with caution by Monaco TPS users when isodose volumes smaller than 1 cm^3^ are involved, due to the inherently increased uncertainty in the fit. GI emerged as the most sensitive DVH parameter to DG variations, while the D_95_ was the least influenced by the DG size, showing clinically irrelevant differences of less than 5%^35^ even for the smallest PTV size considered. Results of this work revealed that D_95_ was the most robust parameter against the spatial discretization errors as expected, since the impact of dose discretization is anticipated to be more severe when calculating isodose volumes rather than when determining dose values confined to individual voxels. Despite the 3‐mm DG appearing to be a reasonable choice for the 30 mm diameter PTVs, percentage differences between the 3‐ and 1‐mm DG‐based DVH indices related to isodose volume calculations exceeded the clinically acceptable tolerance of 5%,^35^ even for the largest PTVs investigated, showing a severe dosimetric consequence of the coarse dose distribution discretization when high conformity to the target and steep dose gradients are evident. Conversely, a 2‐mm DG could be used as an alternative to 1‐mm DG when PTV diameters exceed 20 mm, as it provides an optimal compromise between computational time and dose calculation accuracy. It should be noticed that the 1‐mm DG resulted in approximately 14 and 20 times longer computational times compared to the 2‐mm and 3‐mm DGs, respectively.

Using the ground truth DICOM files, Monaco TPS demonstrated an accuracy within 8.0% and 23.7% in calculating structure and isodose volumes for the 3‐mm target diameters, while an accuracy within 4.9% was achieved for the larger spheres. These findings outperformed the results presented by Stanley et al.^9^ for Eclipse (Varian Medical Systems, Palo Alto, California, USA), Raystation (Raysearch, Stockholm, Sweden) and Velocity (Varian Medical Systems, Palo Alto, California, USA), where percentage differences relative to nominal values for the 3‐mm target using the 1‐mm ST were up to 33.2%, 21.0%, and 15.1%, respectively, for the structure volumes and up to 24.5%, 27.3%, and 36.3%, respectively, for the isodose volumes. The same applies to the larger targets investigated, where Eclipse, Raystation and Velocity demonstrated an accuracy within 9.5%, 6.0%, and 5.9%, respectively. Conversely, Monaco TPS presented an overall accuracy comparable to Elements (BrainLab, Munich, Germany) and MIM (MIM software, Cleveland, Ohio, USA) in calculating structure and isodose volumes across all the considered target spheres. The inconsistencies observed among the different systems may be driven by the method used by each TPS to handle the structure end‐capping and interslice interpolation, as well as by the degrees of supersampling employed by each system. Specifically for supersampling, the Monaco TPS calculates isodose volumes by dividing CT voxels larger than 1 mm^3^ into equal sub‐voxels until each sub‐voxel is smaller than 1 mm^3^. The sub‐voxel is then further subdivided into “mini‐voxels” with their dimensions depending on the grid size, such that the volume of the mini‐voxel is at least 27 times smaller than the volume of the dose‐voxel. The majority of the clinical platforms used by Ma et al.^18^ overestimated the volumes of the considered spheres by up to 20% compared to their known physical values, which does not agree with the findings of our work, yet the contours in their study were generated on a single SRS platform and then exported to the other systems, which may have introduced systematic bias into the results.

Results of this work regarding the impact of ST on dosimetric accuracy can only be quantitatively compared to those of Srivastava et al.^17^ due to the lack of relevant studies in the literature, although their work focuses on intensity‐modulated radiation therapy (IMRT) applications where larger target volumes along with shallower dose gradients are involved compared to SRS applications. Results by Srivastava et al.^17^ for the V_95%_ of a 9.4 cm^3^ cylinder that constitutes the target remained almost constant between the 1 and 2‐mm STs when the 1‐mm DG size was considered, in accordance with findings of our work for the V_100%_ of the larger PTVs, where the corresponding differences in the median values where within 1%. Nelms et al.^7^ suggest that, a decrease in the ST of PINNACLE TPS improves DVH accuracy for small structures more effectively than a reduction in the calculation grid, in contrast to the results of our work for Monaco TPS. Results on the impact of DG on mean, minimum and maximum target doses in VMAT SRS applications of brain metastatic lesions by Pappas et al.^20^ and Han et al.^21^ agree with findings of our work, showing that DVH alteration is proportional to DG size. Karen et al.^15^ observed that, calculation time for the 1‐mm DG increased by 61 and 84% compared to 1.5 and 2.5‐mm DGs, respectively, which was even more pronounced in our work.

To the best of our knowledge, this is the first study validating the DVH capabilities of Monaco TPS for SRS applications. The method developed in this work was proved sensitive in identifying volumetric and dosimetric inaccuracies of Monaco TPS within the context of SRS, that could significantly compromise plan quality and clinical outcome, especially when target diameters are smaller than 20 mm. Given the lack of specific guidance and recommendations for validating the DVH functionality of a new SRS system, this method can be adopted in clinical settings to assist medical physicists in commissioning the DVH of a new TPS. Additionally, it was deemed suitable for establishing clinically relevant thresholds for the ST and DG across various target sizes, optimizing both time efficiency and accuracy. However, it should be noticed that the use of a single TPS constitutes a limitation of this work, rendering the suggested recommendations TPS‐specific. Given the high variability in DVH calculation methodologies across TPSs however,^6^ consensus recommendations on this topic may not be applicable. Another limitation of this study is that it was solely conducted for targets relevant to SRS applications, without investigating the effect of discretization on critical organs dosimetry. Future research could explore this effect using analytical geometries that include critical organs beyond targets, as well as real clinical cases of SRS applications. Such studies would enable users to establish appropriate discretization limits for their systems in order to accurately assess the potential toxicity in critical organs or for decision‐making purposes in scenarios such as re‐irradiation. Additionally, while the impact of ST variations on image registration and contour propagation accuracy in SRS applications is beyond the scope of this work, future evaluation is warranted to provide the radiotherapy community with guidance on how to efficiently integrate image registration in SRS applications.

## 5 CONCLUSION

A method was developed in this work for evaluating the impact of spatial discretization on DVH calculation accuracy in intracranial SRS applications using relevant ground truth data and clinical cases with target diameters ranging from 6 to 30 mm. This method was applied to Monaco TPS offering a better understanding of how structure and isodose volumes interfere with the CT slice thickness and dose grid in the presence of small target volumes and steep dose gradients. Although Monaco TPS is not specifically designed for SRS treatments, it demonstrated an exceptional performance with the 1‐mm ST and DG for both analytical and clinical cases, while DG variations in the clinical cases appeared to dominate discretization errors compared to ST alterations. In the clinical cases, these errors became more pronounced as the isodose volume decreased, while results of the calculated DVH indices revealed that GI and PI were more sensitive to ST and DG changes compared to dose and volume‐based parameters. The 3‐mm DG‐based DVH indices exhibited clinically significant differences for each PTV size compared to 1‐mm DG, while the 2‐mm DG proved to be a reasonable alternative for PTV diameters larger than 20 mm, providing an ideal compromise between calculation time and dosimetric accuracy. Although the DVH indices calculated with the 1.5 and 2‐mm STs were generally statistically equivalent to those calculated with the 1‐mm ST, clinically relevant differences were observed in selected cases with small lesions, suggesting adherence to the 1‐mm ST unless the PTV diameters surpass the 20 mm, where the 1.5 or 2 mm STs can be also considered to minimize the volume delineation time. The method developed in this study for quantifying the effect of spatial discretization on clinically relevant DVH indices could serve as a quality assurance testing procedure for assessing DVH functionality of SRS platforms, allowing end‐users to establish clinically acceptable thresholds for their own systems.

## AUTHOR CONTRIBUTIONS

Conceptualization, Vasiliki Peppa and Pantelis Karaiskos: methodology, Ioanna Grammatikou, Alexandra Drakopoulou, Antigoni Alexiou, Vasiliki Peppa, and Pantelis Karaiskos.: software, Ioanna Grammatikou, Alexandra Drakopoulou, Antigoni Alexiou, and Vasiliki Peppa: validation, Vasiliki Peppa and Pantelis Karaiskos: formal analysis, Ioanna Grammatikou, Alexandra Drakopoulou, Antigoni Alexiou, and Vasiliki Peppa: investigation, Ioanna Grammatikou, Alexandra Drakopoulou, Antigoni Alexiou, and Vasiliki Peppa.: resources, Georgios Pissakas; data curation, Ioanna Grammatikou, Alexandra Drakopoulou, and Vasiliki Peppa: writing—original draft preparation, Vasiliki Peppa: writing—review and editing, Pantelis Karaiskos: visualization, Vasiliki Peppa and Pantelis Karaiskos: supervision, Vasiliki Peppa and Pantelis Karaiskos: project administration, Vasiliki Peppa and Pantelis Karaiskos: All authors have read and agreed to the published version of the manuscript.

## CONFLICT OF INTEREST STATEMENT

The authors have no relevant conflicts of interest to disclosure.

## ETHICS STATEMENT

This retrospective study was approved by the Scientific Review Board of the General Hospital of Athens “Alexandra” (protocol code 644/19‐09‐2024 and date of approval: 25‐09‐2024).
